# TransFactor—prediction of pro-viral SARS-CoV-2 host factors using a protein language model

**DOI:** 10.1093/bioinformatics/btaf491

**Published:** 2025-09-10

**Authors:** Yang An, Valter Bergant, Samuele Firmani, Corinna Grünke, Batiste Bonnal, Alexander Henrici, Andreas Pichlmair, Benjamin Schubert, Annalisa Marsico

**Affiliations:** Computational Health Center, Helmholtz Center Munich, Neuherberg 85764, Germany; School of Computation, Information and Technology, Technical University of Munich, Munich 80333, Germany; Institute of Virology, Technical University of Munich, Munich 80333, Germany; Department of Molecular Biology and Nanobiotechnology, National Institute of Chemistry, Ljubljana 1000, Slovenia; Computational Health Center, Helmholtz Center Munich, Neuherberg 85764, Germany; School of Computation, Information and Technology, Technical University of Munich, Munich 80333, Germany; Institute of Virology, Technical University of Munich, Munich 80333, Germany; Institute of Virology, Technical University of Munich, Munich 80333, Germany; Institute of Virology, Technical University of Munich, Munich 80333, Germany; Institute of Virology, Technical University of Munich, Munich 80333, Germany; German Center for Infection Research (DZIF), Munich Partner Site, Munich 81675, Germany; Systems Virology, Helmholtz Center Munich, Neuherberg 85764, Germany; Computational Health Center, Helmholtz Center Munich, Neuherberg 85764, Germany; Computational Health Center, Helmholtz Center Munich, Neuherberg 85764, Germany

## Abstract

**Motivation:**

Recent pandemics have revealed significant gaps in our understanding of viral pathogenesis, exposing an urgent need for methods to identify and prioritize key host proteins (host factors) as potential targets for antiviral treatments. *De novo* generation of experimental datasets is limited by their heterogeneity, and for looming future pandemics, may not be feasible due to limitations of experimental approaches.

**Results:**

Here, we present TransFactor, a computational framework for predicting and prioritizing candidate host factors using only protein sequence data. It leverages the pre-trained ESM-2 protein language model, fine-tuned on a limited set of experimentally determined host factors aggregated from 33 independent SARS-CoV-2 studies. TransFactor outperforms machine and deep learning baselines and its predictions align with Gene Ontology enrichments of known host factors, but also provide interpretability through a computational alanine scan, enabling the identification of pro-viral protein domains such as COMM, PX, and RRM, that may be used to direct experimental investigations of virus biology and guide rational design of antiviral therapies. Our findings demonstrate the potential of transformer-based models to advance host factor prediction, providing a framework extendable to orthogonal input modalities and other infectious diseases, enhancing our preparedness for current and future viral threats.

**Availability and implementation:**

Source code is available at https://github.com/marsico-lab/TransFactor. A full reproducibility package, including code, trained models, and data, is archived on Zenodo (https://doi.org/10.5281/zenodo.16793684).

## 1 Introduction

Recent pandemics and epidemics, including 2016 Zika, COVID-19, and 2022/23 Mpox, underscore the need to expand our understanding of molecular events governing viral infections. This gap continues to hinder the development of effective antiviral treatments, exposing critical vulnerabilities in our preparedness and responses to both current and emerging viral threats.

Modern molecular biology allows us to study the biochemical basis of viral infections and diseases (Scaturro *et al.* 2018, Stukalov *et al.* 2021, Huang *et al.* 2024), and tackle the three key questions in the field: (i) what are the mechanisms driving disease pathogenicity, (ii) how can disease severity be predicted across a broad spectrum of patients, and (iii) how can disease progression be pharmacologically targeted? Due to their limited protein-coding capacity, viruses rely on the activity of distinct sets of host proteins, termed host factors, to drive aspects of their life cycle, such as uptake, replication, and egress. While most antivirals directly engage viral targets (De Clercq and Li 2016), pharmaceutical inhibition of host factors represents an attractive and under-researched opportunity (Kaufmann *et al.* 2018). However, host factors, and in particular key motifs driving their pro-viral activity, remain largely unknown for most viruses.

Virus host factor identification is dominated by small interfering RNA knock-down, and CRISPR-Cas9 knock-out screens. However, these high-throughput assays suffer from severe drawbacks, such as poor correlation between independent screens, limited availability of suitable cell lines, and variability among them, leading to substantial false-positive and false-negative rates (Baggen *et al.* 2021, Rebendenne *et al.* 2022). Integration and prioritization of findings from high-throughput approaches, augmented with orthogonal information, may be an attractive approach to increase the identification of host targets for antiviral purposes and disease research in general. We envision that such methodologies would systematically increase the utility of high-throughput approaches by guiding experimental validation and drug target assessment efforts.

Computational methods are an emerging field with immense potential to accelerate virus research, including the identification of viral strains that harbor the risk of becoming dominant in the future (Li *et al.* 2024, Rancati *et al.* 2024) and prediction of host proteins crucially involved in viral disease pathogenesis. The latter employ diverse strategies, ranging from the analysis of transcriptomic data comparing control and infected samples, followed by differential gene and isoform expression analysis (Ferrarini *et al.* 2021, Mosharaf *et al.* 2022), to approaches leveraging protein structure to predict host proteins that may physically interact with viral proteins (Tiwari *et al.* 2022). Network-based techniques include computational interrogation of the virus–host protein interaction network to identify key hubs or functionally connected subnetworks (Ravindran *et al.* 2022, Samy *et al.* 2022). Another class focuses on predicting subtypes, such as RNA-binding proteins that interact with viral RNA. These predictions utilize bioinformatics pipelines or machine learning models (Vandelli *et al.* 2020, Horlacher *et al.* 2023). Finally, we and others in the past successfully used graph-based approaches to integrate knowledge on host biology with multi-omics profilings to prioritize functional follow-up of hot spots of cellular signaling perturbations upon virus infections, as well as to repurpose existing drugs toward potential SARS-CoV-2 drug targets (Morselli Gysi *et al.* 2021, Ruiz *et al.* 2021, Stukalov *et al.* 2021, Bergant *et al.* 2022, Huang *et al.* 2024). Many of these methodologies rely on graph-based assemblies of the host protein functional interaction landscape, such as STRING (Szklarczyk *et al.* 2023), which are often based on data mining and can therefore be prone to noise. These graphs are often highly connected, despite only distinct interactions being functional and impactful in any given biological state. Their undirected nature further introduces erroneous information aggregation as the causal direction of the interaction is not accounted for. Random walk with restart is then commonly applied, which assumes a linear combination of individual mechanisms, neglecting synergistic or antagonistic effects. Moreover, they are heavily reliant on omics measurements of *in vitro* virus infection systems, which are challenging to characterize, especially for emerging viruses, and show a high degree of variability between them. Collectively, assessment of alternative data modalities and suitable models is urgently needed to improve host factor identification in real-world scenarios and to consolidate our preparedness for existing and future viral threats.

Transformer-based protein language models (PLMs) have significantly advanced protein biology [comprehensive overview in Wang *et al.* (2025) and Xiao *et al.* (2025)]. Trained on large protein datasets in a self-supervised manner, these models learn to extract meaningful contextual, local, and global sequence features. Fine-tuning these models has enabled accurate predictions of various protein attributes, including function, fitness, family classification, and structure (Schmirler *et al.* 2024). Recently, PLMs have been fine-tuned or fully trained specifically on viral proteins to predict escape mutations, potentially arising strains, and to design prospective vaccines (Hie *et al.* 2022, Dhodapkar 2023, Thadani *et al.* 2023, Rancati *et al.* 2025, Liu *et al.* 2025, Youssef *et al.* 2025).

In this study, we propose TransFactor, a PLM-based model for predicting pro-viral SARS-CoV-2 host factors using only the protein sequence information, without the need for acquiring additional omics measurements. TransFactor leverages the pre-trained PLM ESM-2 (Lin *et al.* 2023) and significantly outperformed baseline methods in terms of prediction performance, such as SVMs and deep learning models. We further evaluated TransFactor’s ability to generate biologically relevant hypotheses by applying the model to prioritize candidate host factors (with limited experimental evidence in the literature). Our results demonstrated that high-ranking candidates were more enriched than low-ranking ones in molecular functions and processes of the known host factor set. By interpreting the model’s predictions using an alanine scan (Massova and Kollman 1999, Kortemme *et al.* 2004), we identified protein regions or domains most critical for predicting SARS-CoV-2 host factors.

We envision that TransFactor will support both basic and applied antiviral research by ranking and shortlisting candidate proteins for experimental validation, accelerating the identification of host factors and their pro-viral domains, as well as assist in the design of novel antivirals.

## 2 Materials and methods

### 2.1 Data

#### 2.1.1 Human protein sequences and domain information

The human proteome was assembled by collecting all 20 415 canonical and reviewed protein sequences of organism ID 9606 from UniProtKB/Swiss-Prot (accessed 2019.10.08) (uni 2025). Protein domain annotations were retrieved from UniProtKB in December 2024, including the features “Signal peptide,” “Domain,” “Region,” “Zinc Finger,” “DNA binding,” “Motif,” “Active site,” “Binding site,” and “Site.”

#### 2.1.2 Host factor labels

**Figure 1.**
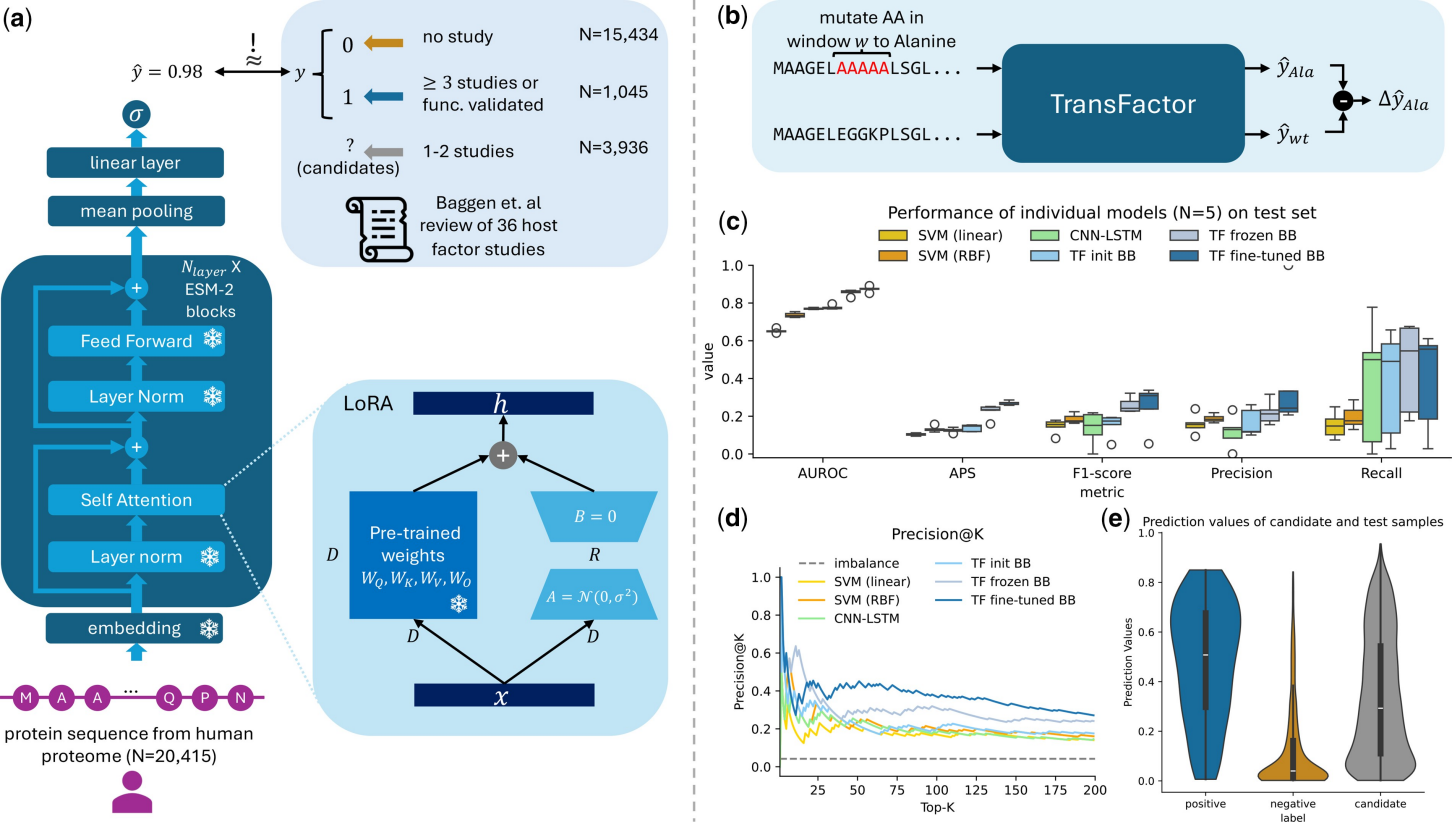
TransFactor framework and performance. (a) A protein sequence from the human proteome is fed into the pre-trained ESM-2 backbone to extract contextual features for each residue. These feature vectors are mean pooled along the sequence length dimension and passed through a linear layer and consecutive sigmoid activation to create a score between 0 and 1. Proteins were labeled based on outcomes from 33 independent studies reviewed by Baggen *et al.* (2021). LoRA (Hu *et al.* 2022) was adapted on the weights within the attention module of ESM-2, while the other parts of the backbone were frozen. (b) Schematics of the computational alanine scan. The wild-type protein sequence is mutated to alanine in a window *w*, and the difference $\Delta {\hat{y}}_{\mathrm{Ala}}$ in prediction between the mutated and wild-type sequence is taken as attribution score of the mutated positions. (c) Performance of individual models on the test set. Each model was tuned and trained on five distinct data splits, and for each split, the hyperparameter configuration yielding the highest AUROC was selected for further evaluation. As baselines, SVMs similar to Bressin *et al.* (2019) with linear and radial basis function kernel, a CNN-LSTM hybrid model similar to Wu and Guo, (2024), and TransFactor (TF) with backbone randomly initialized (init BB) and frozen (frozen BB) were used. (d) Precision@K on the Top-K predicted proteins by each model. (e) Violin plot of prediction values for positive and negative test samples as well as candidate samples using TransFactor ensemble with ESM-2 backbone.

We labeled the proteins by aggregating the results from 33 independent SARS-CoV-2 assays, encompassing genome-wide, arrayed, and targeted functional screens, as well as interactomics studies, reviewed by Baggen *et al.* (2021) (Fig. 1a). Proteins with at least three corroborating high-throughput studies, or at least one low-throughput functional study, were considered as positives (*N* = 1045 host factors). Conversely, proteins absent in any study were considered as negatives (*N* = 15 434). Importantly, this way of classifying proteins was chosen due to the inherently noisy nature of high-throughput studies, which results in a minimal overlap between significant hits originating from independent studies (Baggen *et al.* 2021). This is further compounded by the use of different experimental systems, i.e. cell lines, statistical tests, time points, and infection doses. Based on this and our prior experience with similar assays, we expected the data to contain a significant proportion of false positives and false negatives. Finally, proteins found to be potential host factors by one or two studies were considered as candidate host factors (*N* = 3936). These were not used during training or performance evaluation. One aim of this study was to rank the candidate set according to their likelihood of playing a pro-viral role during SARS-CoV-2 infection, thereby generating hypotheses for validation in low-throughput functional assays.

### 2.2 Model architecture

In this work, we developed and trained a model *M* that takes in a protein sequence *X* and predicts a score indicating whether the sample is a host factor $\hat{y}(X)=M(X)$. Let $X=\{x_{1},x_{2},\ldots ,x_{L}\}$ be a sequence of length *L*, where each residue $x_{i}\in AA=\{A,C,\ldots ,Y\}$ is from the set of the 20 canonical amino acids (*AA*) (Fig. 1a).

We used ESM-2 (Lin *et al.* 2023), an encoder-only transformer (Vaswani 2017) PLM that has been pre-trained on 65 million unique protein sequences using the masked language modeling task (Devlin 2019). The contextual residue features $H=\mathrm{ESM}2(X)\in R^{L\times D}$ with a hidden dimension *D* after the last transformer layer were extracted. Due to quadratic memory scaling, we used the first 1024 residues, which 90% of our input proteins do not exceed. The mean pooled fixed-length sequence-wise feature vector $h_{p}\in R^{D}$ was then fed into a linear layer with a consecutive sigmoid layer to gain a scalar host factor score $\hat{y}$ between 0 and 1.

The model was trained in a classification setting to distinguish host factors (positives) from non-host factors (negatives), optimizing the binary cross-entropy loss. Due to class imbalance, we scaled the loss of positive samples by a factor λ, which was treated as a hyperparameter to be optimized during tuning.

### 2.3 Training procedure

The dataset was split into six folds; five were used for cross-validation, while the sixth fold was held out as a test set for final evaluation. To prevent data leakage, we used mmseqs2 (Steinegger and Söding 2017) to cluster similar sequences. Since the human proteome contains mostly dissimilar proteins, the parameters were chosen lower than the minimum sequence identity of 50% commonly used for the pre-training of PLMs. The following command and parameters were used:

*mmseqs easy-cluster sequences.fasta cluster_dir tmp -c 0.1 –min-seq-id 0.1 -e 0.001*

**Figure 2.**
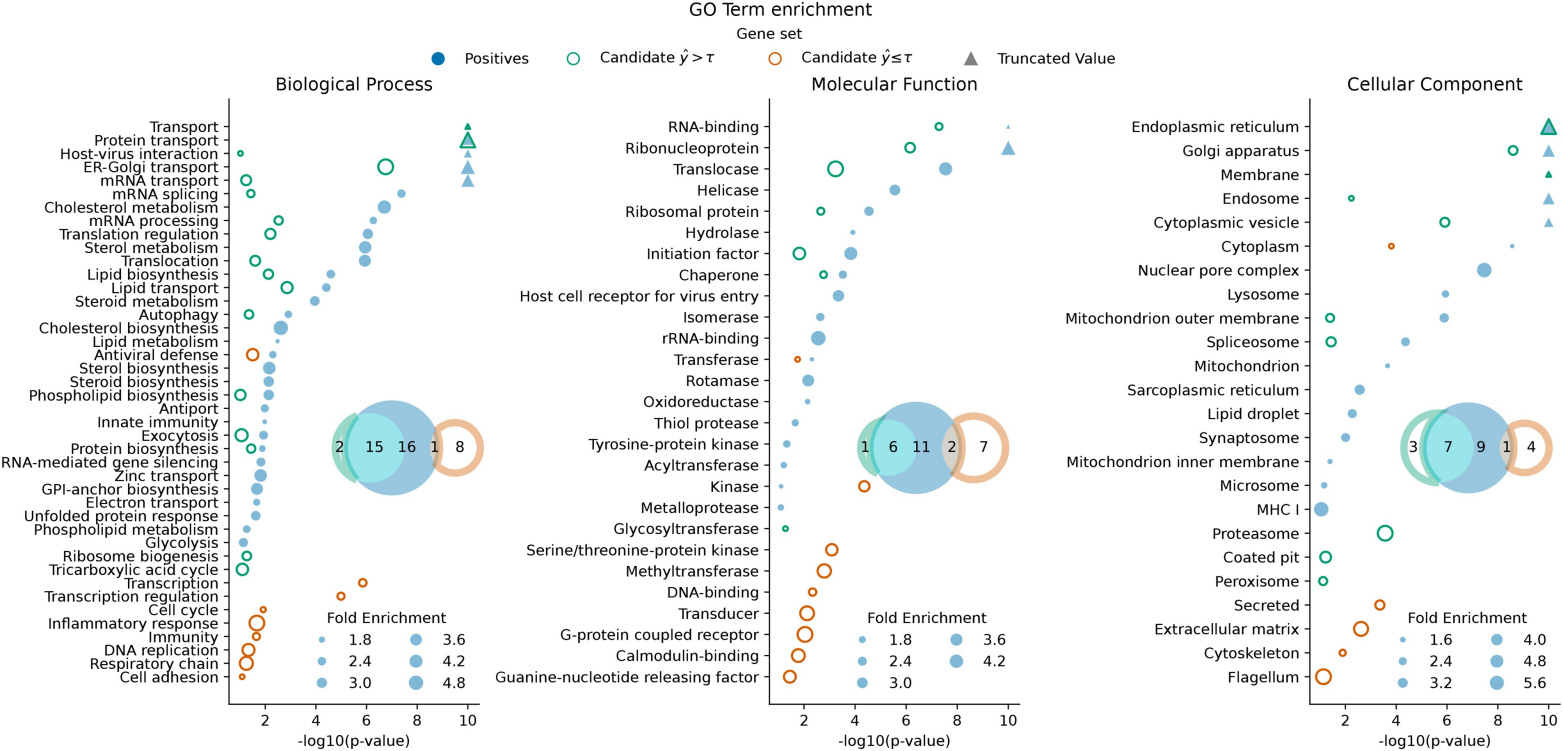
GO-term enrichment was performed on the positively labeled proteins (using all proteins as background), and on the candidate proteins (taking all candidate proteins as background) predicted as positive ($\hat{y}>\tau$) and negative ($\hat{y}\leq \tau$). The enrichment analysis was performed separately for all three gene ontologies, and terms significant for at least one gene set are displayed. Venn diagrams show the overall and overlapping number of enriched terms in and between the three sets.

**Figure 3.**
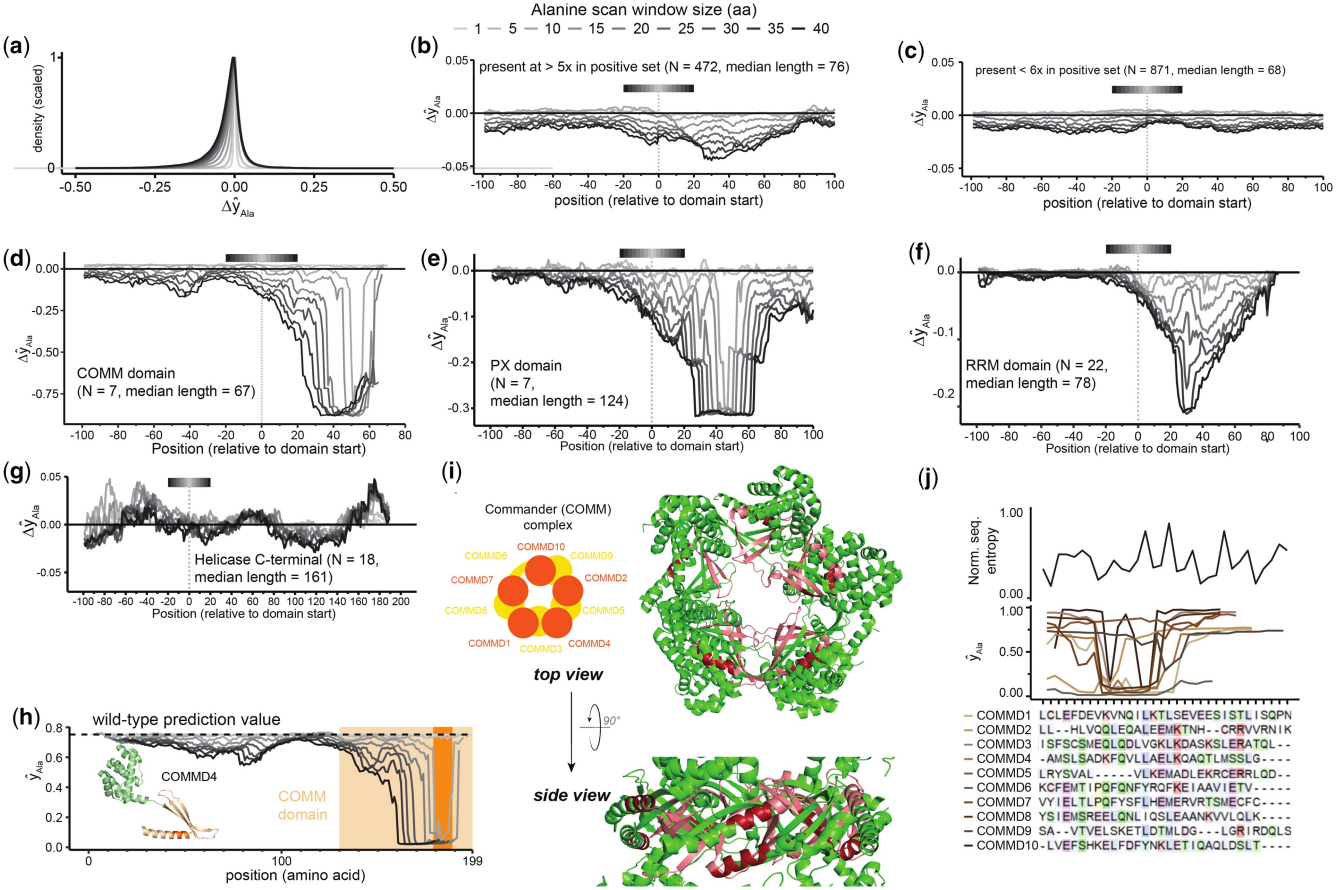
Computational alanine scans were performed using indicated amino acid window sizes for all positives. (a) Density plot depicting $\Delta {\hat{y}}_{\mathrm{Ala}}$ attribution values. (b, c) Domains that were present more, (b) or less, or equal (c) than five times in the positive samples, were used for depicting the median $\Delta {\hat{y}}_{\mathrm{Ala}}$ with respect to amino acid positions relative to the domain start. (d–g) Similar to (b, c), but only the three domains with the highest absolute average $\Delta {\hat{y}}_{\mathrm{Ala}}$ attribution scores—COMM (d), PX (e), RRM (f)—and a domain with neutral $\Delta {\hat{y}}_{\mathrm{Ala}}$—Helicase C-terminal (g)—were used. (h) Prediction scores for protein COMMD4 (protein structure PDB: 8F2R). Light orange—COMM domain; dark orange—amino acid positions, where prediction values are below 0.4 for all alanine scan window sizes. (i) Structure of the 10-protein complex consisting of COMMD1-10 (PDB: 8F2R)—regions where $\Delta {\hat{y}}_{\mathrm{Ala}}<-0.5$ for alanine scan window size of 40 are highlighted in pink; similarly, regions in which this condition is satisfied for alanine scan window size of 10 are further highlighted in red. (j) Bottom: amino acid sequences of proteins COMMD1–10 were aligned using ClustalOmega (Madeira *et al.* 2024). Top: alanine scan prediction values (${\hat{y}}_{\mathrm{Ala}}$) were corrected according to the depicted alignment and plotted for the indicated proteins (alanine scan window size of 10) alongside the 10-letter amino acid alphabet normalized entropy (0 indicates low conservation, 1 indicates high conservation). Amino acids corresponding to the COMMD4 positions 172–199 (panel h, dark orange) are shown. Unless otherwise specified, individual panels reuse color coding from panel (a).

Members of each cluster were grouped into a single fold. Almost half the proteins were assigned to clusters with sizes smaller than six, and 21% were singleton clusters. When taking the most abundant Gene Ontology (GO)-term from each cluster, the resulting non-singleton clusters had an average GO-term purity of 82%, 90%, and 88% for biological process, cellular component, and molecular function, respectively. These results indicate that proteins were grouped into functionally similar clusters, therefore effectively reducing the risk of data leakage (Figs 2 and 3, available as supplementary data at *Bioinformatics* online). Additionally, the splits were stratified by their label to approximately balance the ratio of positives and negatives between the splits.

Hyperparameter optimization was conducted using Optuna (Akiba *et al.* 2019), maximizing AUROC as selection criterion. On each split, hyperparameter optimization was performed for 48 h on a machine with an Nvidia A100. Early stopping was done after 25 epochs without improvement on the criterion. The model from each split with the highest validation AUROC was used for performance evaluation and downstream analysis. To aggregate the prediction values and improve the stability and performance, we further used the five resulting models in an ensemble mode by taking the average prediction score. We opted for averaging as the models share the same base architecture, while differing in their training data splits. Hence, we assume that applying the same weighting provides a stable consensus.

To prevent catastrophic forgetting and improve the training speed and memory requirements, we used Low-Rank Adaptation (LoRA) (Hu *et al.* 2022) to fine-tune the ESM-2 backbone. Following the original paper, we only adapted the attention weights $W_{Q},W_{K},W_{V},W_{O}$, while freezing all other parameters of the ESM-2 backbone.

### 2.4 Baselines and ablation

We evaluated TransFactor against two baselines and two ablation variants. First, we re-implemented TriPepSVM (Bressin *et al.* 2019)—a linear and RBF-kernel SVM that classifies proteins based on overlapping 3-mer counts. Second, we adapted a CNN-LSTM hybrid similar to Wu and Guo (2024), which uses the protein sequence as input and passes the last hidden features of the LSTM as input into a linear classification head. Finally, to assess transfer learning, we trained two ablated versions of TransFactor, one with frozen ESM-2 weights (TF frozen BB), and one with a randomly initialized backbone rather than pre-trained (TF init BB). For details of baselines and hyperparameters, refer to Text A.1 and Tables 2–6, available as supplementary data at *Bioinformatics* online.

### 2.5 GO enrichment

GO enrichment analysis was conducted using the Database for Annotation, Visualization, and Integrated Discovery (DAVID) tool (DAVID Knowledgebase v2024q4, released on 22 December 2024; available at https://davidbioinformatics.nih.gov/summary.jsp) (Sherman *et al.* 2022). The analysis was performed separately for the candidate proteins with a prediction score above and below the ideal threshold τ, which was determined on the validation set as reaching the highest *F*1-score. The entire candidate protein set was used as the background. Similarly, for the positive protein set, the entire protein dataset was used as the background. Enrichment was assessed across the three main GO categories: biological process (BP), molecular function (MF), and cellular component (CC). Default parameters in DAVID were applied for statistical testing and multiple testing correction.

### 2.6 Model’s interpretation through computational alanine scan

To understand the attribution of amino acid motifs to the overall prediction, we performed a computational alanine scan (Massova and Kollman 1999, Kortemme *et al.* 2004) (Fig. 1b). For the selected protein sequence *X*, we substituted the amino acids in a contiguous window with alanine. Alanine has a small methyl side chain and, therefore, is the most functionally inert amino acid, commonly used in single-point-mutagenesis-based experiments to interrogate the functional relevance of distinct protein regions (Morrison and Weiss 2001). We utilized window sizes *w* of 1, as well as values ranging from 5 to 40 in increments of 5. The wild-type sequence was mutated to $X_{x_{i:i+w}\to A}=\{x_{1},\ldots ,x_{i-1},A,\ldots ,A,x_{i+w},\ldots ,x_{L}\}$. We then used the trained model to predict the host factor score $\hat{y}(X_{x_{i:i+w}\to A})$ of the mutated sequence. The difference in prediction score $\Delta {\hat{y}}_{\mathrm{Ala}}=\hat{y}(X_{x_{i:i+w}\to A})-\hat{y}(X_{wt})$ between the mutated and wild-type sequence was used as the attribution score for residues within the substitution window. For samples from the test set, we used the ensemble model prediction scores, while for samples from the cross-validation splits, the model corresponding to the validation set was used.

For domain-wise statistical testing of significant deviations of $\Delta {\hat{y}}_{\mathrm{Ala}}$ (Fig. 4, available as supplementary data at *Bioinformatics* online), we used the one-sided Wilcoxon rank-sum test and compared $\Delta {\hat{y}}_{\mathrm{Ala}}$ values within domains to all values across all proteins for any given alanine scan window. The thus obtained *P*-values were further FDR-adjusted.

## 3 Results

### 3.1 TransFactor outperforms baseline models in predicting SARS-CoV-2 host factors

First, we evaluated the performance of our proposed method on the held-out test set. Due to the high imbalance of 4% positive test samples, we chose the area under the receiver operating characteristics curve (AUROC), average precision score (APS) as a conservative estimation method for the area under the precision–recall curve, and *F*1-score, further broken down into precision and recall as metrics. We determined the ideal thresholds τ based on the highest *F*1-score on the validation set for each model, respectively. For the ensembles, we optimized the threshold on the whole training dataset (τ=0.571). The benchmark results of the best model trained on each of the five folds are shown in Fig. 1c and Table 1 (first row within each method), available as supplementary data at *Bioinformatics* online.

The simplest model, an SVM with a linear kernel, consistently showed the lowest performance across all metrics. Replacing the linear kernel with an RBF kernel led to moderate improvements. Both sequential deep learning models, the CNN-LSTM hybrid and TransFactor with a randomly initialized backbone, performed similarly to SVM with RBF kernel. However, using a frozen pre-trained ESM-2 backbone greatly increased AUROC (0.78–0.86), APS (0.14–0.24), and *F*1-score (0.19–0.30) in comparison to the best other model. Using LoRA fine-tuning on the weights of the backbone, we could further improve the performance in three out of five metrics.

These results highlight the need for pre-trained language models for these data. The sequential deep learning models failed to outperform the traditional machine learning baseline. This indicates that the sequential models could not identify and extract functional information from the raw sequences. One possible explanation lies in the diversity of the human proteome. Sequences originated from diverse sets of protein families with very high dissimilarities to each other. Specifically, the 20 415 sequences were distributed in 6961 clusters despite a minimum sequence identity of 10% and coverage of 10%. Forty-eight of all sequences were in clusters of size five or smaller. These properties make it challenging for the model to rely solely on sequence information. Through pre-training, the model learns to extract meaningful features and capture functional information from evolutionary conservation patterns, where distant sequences may still result in related function and structure.

Next, the prediction scores of the five individual models were aggregated and averaged. The resulting ensemble models consistently improved upon the mean performance for all individual models of each underlying architecture (Table S1, second row within each method, available as supplementary data at *Bioinformatics* online). For TransFactor with fine-tuned backbone, we observed increases in AUROC from 0.87 to 0.89, APS from 0.27 to 0.30, and *F*1-score from 0.23 to 0.38 through the aggregation of prediction scores. To gain an estimate of the expected hit rate during validation, we calculated the Precision of the Top-K predicted samples (Precision@K) (Fig. 1d). Except for the noisy lower K range, TransFactor consistently reached higher Precision@K values than the baseline methods. At typical experimental capacities of 50, 100, and 200, TransFactor had a precision of 0.44, 0.37, and 0.28, respectively. Due to the condensed score and higher performance, we used the ensemble model for further analyses, unless otherwise indicated.

To further contextualize the performance of our computational method, we evaluated the predictive power of experimental screens by using each one (excluding functional validation screens) from the review by Baggen *et al.* (2021) to correctly identify host factors from the full human proteome. We applied our labeling scheme, with a minor modification: the screen under evaluation was excluded from the labeling process. On average, experimental screens achieved an *F*1-score of 0.13 ± 0.11 (mean ± standard deviation), with a precision of 0.60 ± 0.25 and a recall of 0.11 ± 0.13 (details in Table S7, available as supplementary data at *Bioinformatics* online).

### 3.2 GO-term enrichment reveals biological consistency in high-scoring uncertain proteins

To assess the potential of our model in guiding the selection of protein candidates from high-throughput screens with lower precision, we scored each protein from the candidate set with TransFactor. The resulting score distribution fell between those of the positive and negative test samples (Fig. 1e). Eight hundred eighty-three of the 3936 candidates were predicted as potential host factors. High-throughput screenings are expected to yield many false positives, consistent with our prediction’s distribution. This indicates TransFactor’s potential to help distinguish prospective novel host factors from experimental noise, proposing a shortlist of candidates for further investigation.

Next, we evaluated TransFactor’s predictions using GO enrichment analysis to determine whether the model captures biological relevance and stratifies candidate proteins into promising and less promising ones. Enriched GO-term of predicted host factors closely mirrored those of known positives, showing strong overlap across BP, MF, and CC categories [Fig. 2; Fisher’s exact test: BP (OR = 15.0, *P*-value = .0001), MF (OR = 9.23, *P*-value = .0395), and CC (OR = 4.67, *P*-value = .0663)]. By contrast, negatively predicted candidates exhibited little overlap [BP (OR = 0.16, *P*-value = .0780), MF (OR = 0.44, *P*-value = .4582), and CC (OR = 0.32, *P*-value = .6323)).

These results indicate that the model successfully captures the structural and functional organization of host factor proteins. This predictive capability could provide a valuable framework for prioritizing putative candidates for targeted experimental validation.

### 3.3 Computational alanine scan identifies domains important for the model’s prediction

To identify motifs and domains that were affecting the predictions, we performed a computational alanine scan on the positive subset of proteins (Massova and Kollman 1999, Kortemme *et al.* 2004). We employed a broad range of window sizes to introduce varying degrees of perturbations to protein sequences, enabling us to assess the impact of both small- and large-scale changes on prediction scores. This approach allowed us to find a balance between the amplitude and resolution of the explanations.

First, we evaluated whether the model accurately captured the well-established principle that substituting amino acids with alanine often reduces protein functionality. Consistent with this concept, our results revealed a clear trend of decreased prediction scores following alanine substitutions. Notably, this effect became more pronounced with increasing sizes of the alanine scan windows (Fig. 3a).

Furthermore, we mapped the alanine scan attribution scores to protein domains to assess if the model learned to specifically penalize the alanine substitutions in regions of proteins known to be functionally relevant. Notably, we observed a decrease in median attribution scores within protein domains enriched in the positive subset (Fig. 3b and Fig. 4a, available as supplementary data at *Bioinformatics* online). In contrast, domains less represented in positively labeled proteins showed almost no decrease in median attribution scores (Fig. 3c and Fig. 4a, available as supplementary data at *Bioinformatics* online). These findings strongly indicate that the model can distinguish protein sequences and regions that are more important for host factor classification and can learn from given exemplary sequences containing similar regions.

After showcasing the general prevalence of the model to recognize the functionally important features of proteins, we took a detailed look at some domain types. Importantly, not all domains exhibited statistically significant decreases in their attribution scores in comparison to values across all scanned proteins (Fig. 4b, available as supplementary data at *Bioinformatics* online). While we observed the strongest decrease upon introduction of alanine substitutions in copper metabolism gene MURR1 (COMM) domains (Fig. 3d), phox homology (PX) domains (Fig. 3e), and RNA recognition motifs (RRM) (Fig. 3f), we did not observe this for many other domains such as Helicase C-terminal domains (Fig. 3g). Particularly striking was the strong decrease in attribution scores for the COMM domains of COMMD proteins. In humans, there are 10 COMMD proteins, which encode a COMM domain. Seven of them were previously shown to play a role in SARS-CoV-2 infection (Zhu *et al.* 2021) and thereby were contained in our positive set (COMMD2/3/4/5/7/8/10) (Baggen *et al.* 2021). COMMD proteins, together with CCDC22 and CCDC93, form the CCC complex, which, together with the retriever complex (VPS35L, VPS26C, VPS29), forms the commander complex, involved in the endosomal cargo trafficking and recycling (Healy *et al.* 2023). COMMD proteins, as well as proteins and assemblies related to these processes, were previously shown to be SARS-CoV-2 host factors (Baggen *et al.* 2021), but to the best of our knowledge, no specific parts of these proteins are so far known to be critical for this functionality. COMMD proteins, except COMMD6, were among the top predicted host factors by TransFactor and had prediction scores between 0.75 and 0.98. Interestingly, our alanine scan results, in particular evident for the lower range of alanine scan window sizes, suggested that the relatively poorly conserved C-terminal part of the COMM domain in COMMD4 (Fig. 3h) and other COMMD proteins (Fig. 3i–j and Fig. 5, available as supplementary data at *Bioinformatics* online) may play a central role in their ability to support SARS-CoV-2 replication as host factors.

## 4 Discussion

The COVID-19 pandemic has resulted in unprecedented socioeconomic disruptions and more than 6 million lost lives. Despite the need for effective antiviral therapies, we still do not fully understand the molecular basis of SARS-CoV-2 infection. Virology relies on experimental studies of virus–host interactions, but dataset heterogeneity and practical challenges, such as high virulence or hard-to-culture viruses, limit comprehensive characterization of host factors and their role in infection etiology and progression. High-throughput methods are often infeasible for some viruses or poorly followed up due to resource constraints, underscoring the need for computational models to predict and prioritize key interactions from limited and noisy data. Emerging AI technologies, such as sequence-based deep learning models, offer significant potential to uncover critical host factors that facilitate viral infections. Inspired by the success of PLMs, we developed TransFactor—a transformer-based method for predicting virus host factors based on protein sequence information and a limited set of experimentally determined host factors. By leveraging pre-trained PLM’s feature extraction capabilities and fine-tuning on the classification task of distinguishing SARS-CoV-2 host factors from non-host factors, TransFactor outperformed machine and deep learning baseline methods. Candidate host factors prioritized by TransFactor showed similar GO-term enrichments as known host factors, giving more confidence in the model’s capability to rank and prioritize proteins. Through a computational alanine scan, TransFactor could identify domains important for the prediction, helping to understand the molecular basis underlying host factors.

However, TransFactor faces limitations that present opportunities for improvement in future work. Currently, TransFactor’s input is truncated to 1024 amino acids for efficient training and inference of the model, which may omit important C-terminal regions relevant for host–virus interactions. To assess this, we reevaluated our trained models on the test proteins truncated at 2048, increasing the coverage from 90% to 98%. This yielded a slight improvement in five out of eight performance metrics (Table S8, available as supplementary data at *Bioinformatics* online). Nevertheless, as computational resources grow drastically with sequence length, and practical implications for large proteins (longer than 1000), including reduced sequencing fidelity, expression strength, and detection by Western blot, we adopted the shorter length for this work. To further mitigate these aspects, we plan to explore random cropping during training, memory-efficient methods like FlashAttention (Dao *et al.* 2022), and fine-tuning on longer sequences in the future.

To effectively train and employ deep learning models, an extensive amount of labeled data, i.e. known host factors, is needed. Such data is not universally available for many viruses, originates from a variety of strains, and was acquired in different infection models. However, transfer learning and domain adaptation techniques could leverage data from closely related viruses to make predictions for those with limited or no known host factors. Transferability depends primarily on the similarity of pathways and biochemical processes the viruses need for their life cycles and the resulting set of host factors. The overlap and divergence of essential host processes could give an estimate for the success of the transfer. Yet, for most viruses, these factors are not known in sufficient depth. Instead, the genetic similarity and evolutionary proximity can serve as a proxy. To demonstrate this, we used our TransFactor model trained on SARS-CoV-2 host factors to predict host proteins interacting with SARS-CoV viral proteins as a proxy for its host factors (*N* = 612, Stukalov *et al.* 2021) without fine-tuning. While the performance declined as expected, we were able to recall 42% of SARS-CoV, indicating the potential to prioritize host factors for phylogenetically related viruses. In contrast, the model failed to identify putative host factors (*N* = 368, Montoya *et al.* 2023) of the phylogenetically distant HIV with a recall of 18% (details in Table S9, available as supplementary data at *Bioinformatics* online).

Moreover, TransFactor currently relies exclusively on primary sequence information, which makes classifying proteins with low homology to the training data particularly challenging. Integrating orthogonal data, such as the tertiary structure, may provide the model with additional valuable insights into protein functionality, alleviating the problem of low sequence homology. Similarly, incorporating additional experimental information such as peptide-level abundances, phosphorylation, and ubiquitination events obtained by LC–MS/MS-based proteomics may allow the model to access signaling perturbations instigated by the invading pathogen, allowing it to consider cellular signaling state on one or more functional layers. As proteins rarely act in isolation, incorporating protein–protein interaction network data could add a higher-order layer to the model, capturing complex biological processes. These additional data modalities could help address current challenges, such as low *F*1 scores, by identifying features not captured by sequence information alone and expand the generalizability to related and potentially more distant viruses. Addressing label noise and the high imbalance in existing host factor datasets through further low-throughput functional studies and explicit modeling of label uncertainty could also improve the model’s discriminative performance. Ultimately, experimental validation of TransFactor’s predictions through functional screens will be essential. The results of such experiments could further enhance the model in an active learning, lab-in-the-loop setting. While we presented a proof-of-concept on SARS-CoV-2 host factors, further evaluations on other diseases are necessary to assess the generalizability of TransFactor.

In summary, we have shown that TransFactor can reliably identify and rank host factor proteins. Combined with a computational alanine scan, TransFactor enables the detailed analysis and interpretation of how specific amino acid motifs contribute to pro-viral pathogenesis. This approach provides valuable insights at a meaningful scale, offering a robust foundation for generating hypotheses that can be further tested in appropriate experimental models, facilitating the advancement of our biological understanding of viral infectious diseases, and providing a valuable resource to guide rational antiviral design.

## Supplementary Material

btaf491_Supplementary_Data

## Data Availability

Source code is available at https://github.com/marsico-lab/TransFactor. A full reproducibility package, including code, trained models, and data, is archived on Zenodo (https://doi.org/10.5281/zenodo.16793684).
