# Supplementary material for: TransFactor—prediction of pro-viral SARS-CoV-2 host factors using a protein language model
Source: Bioinformatics. 2025 Sep 10;41(9):btaf491. doi: 10.1093/bioinformatics/btaf491 (PMC12449051; doi:10.1093/bioinformatics/btaf491)
Supplement: btaf491_Supplementary_Data [file btaf491_supplementary_data.pdf]

Supplementary Materials for TransFactor - Prediction of pro-viral SARS-CoV-2 host factors using a protein language model

Yang An,[1,2] Valter Bergant,[3,4] Samuele Firmani,[1,2] Corinna Grünke,[3] Batiste Bonnal,[3] Alexander Henrici,[3] Andreas Pichlmair,[3,5,6†] Benjamin Schubert[1,†] and Annalisa Marsico[1,†,*]

[1]Computational Health Center, Helmholtz Center Munich, Neuherberg 85764, Germany, [2]School of Computation, Information and Technology, Technical University of Munich, Munich, 80333 Germany, [3]Institute of Virology, Technical University of Munich, Munich, 80333 Germany, [4]Department of Molecular Biology and Nanobiotechnology, National Institute of Chemistry, Ljubljana 1000, Slovenia, [5]German Center for Infection Research (DZIF), Munich Partner Site, Munich, 81675 Germany, [6]Systems Virology, Helmholtz Center Munich, Neuherberg, 85764 Germany and [†]equal contribution

[*]Corresponding author. annalisa.marsico@helmholtz-munich.de

A. Supplementary Text

A.1. Baselines and ablation

To compare the performance of TransFactor, we created two baselines and two ablation models. First, we utilized a Support Vector Machine (SVM) similar to TriPepSVM (Bressin et al., 2019) that takes as input 3-mers counts within a protein with overlap, represented as a count vector, to classify protein sequences. We re-implemented TriPepSVM with its original linear kernel as well as a radial basis kernel.

Second, a hybrid between Convolutional Neural Network (CNN) and Long Short-Term Memory (LSTM) (Hochreiter, 1997) model with an architecture similar to (Wu and Guo, 2024) was employed. The original architecture consists of two CNN blocks as feature extractor and an LSTM to aggregate the features. The last hidden feature from the LSTM is passed into a linear layer with a sigmoid activation function. We re-implemented the model to take in the same protein sequences representation as TransFactor up to sequence length 1024, exchanged the position-specific scoring matrix input representation to a learned token embedding layer, and added ReLU as activation after the CNN layer in each block. We further kept the number of CNN blocks tunable and ran the same hyperparameter optimization protocol as for TransFactor for each split.

Lastly, we created two ablated versions of TransFactor. The first version, termed *TF init BB*, where the weights of the ESM-2 backbone were randomly initialized instead of loading pre-trained ESM-2 weights to study the effect of transfer learning. For this version, two full hyperparameter optimization runs were performed on each split with differing model size ranges. The first run had capacities between our used backbones *ESM-2_t12_35M_UR50D* and *ESM-2_t30_150M_UR50D* (embedding dimension between 480 and 640 and number of layers between 12 and 30). As these sizes were prone to overfitting, we further ran hyperparameter optimization for smaller models with embedding dimensions between 16 and 128, as well as the number of layers between 2 to 6. Since the best smaller models slightly but consistently outperformed the bigger models on their corresponding validation set, we report only the performance of the smaller models. Second, to study the effects of fine-tuning, we trained a version of TransFactor with a frozen ESM-2 backbone (*TF frozen BB*), hence effectively only training the classification head on ESM-2 extracted embeddings. Here, we again followed our cross-validation scheme and performed a hyperparameter optimization on each split.

Used hyperparameter sets can be found in Supp. Table 2-6.

© The Author . Published by Oxford University Press. All rights reserved. For permissions, please e-mail: journals.permissions@oup.com

B. Tables

Supp. Table 1. Comparison of performance on the test set. First row for each model shows the average performance with a standard deviation of five individual models, tuned and trained on five different folds. The second row shows the performance when taking the five models as an ensemble. In bold, the best-performing model for each metric is highlighted. TF: TransFactor, BB: Backbone

model	AUROC	APS	F1-score	Precision	Recall	Precision@50	Precision@100	Precision@200
SVM (linear)	0.65±0.01	0.10±0.01	0.15±0.04	0.16±0.05	0.15±0.07	0.18±0.05	0.15±0.03	0.13±0.01
	0.67	0.11	0.16	0.17	0.15	0.20	0.17	0.140
SVM (RBF)	0.74±0.01	0.13±0.02	0.19±0.03	0.19±0.02	0.20±0.06	0.20±0.03	0.19±0.01	0.15±0.01
	0.74	0.14	0.21	0.20	0.23	0.24	0.20	0.160
CNN-LSTM hybrid	0.77±0.00	0.13±0.01	0.14±0.09	0.12±0.09	0.38±0.33	0.18±0.08	0.16±0.02	0.14±0.01
	0.78	0.14	0.21	0.15	0.35	0.24	0.18	0.145
TF init BB	0.78±0.01	0.14±0.02	0.15±0.06	0.17±0.07	0.37±0.29	0.20±0.05	0.16±0.04	0.15±0.02
	0.79	0.16	0.23	0.18	0.30	0.22	0.20	0.175
TF frozen BB	0.86±0.02	0.24±0.04	**0.30±0.04**	0.23±0.05	**0.45±0.09**	0.28±0.06	0.24±0.05	0.22±0.03
	0.87	0.25	0.31	0.23	**0.47**	0.30	0.31	0.24
TF fine-tuned BB	**0.87±0.01**	**0.27±0.01**	0.25±0.12	**0.40±0.34**	0.39±0.27	**0.37±0.03**	**0.32±0.02**	**0.26±0.01**
	**0.89**	**0.30**	**0.38**	**0.34**	0.44	**0.44**	**0.37**	**0.275**

Supp. Table 2. Hyperparameters of TransFactor model with best AUROC in the corresponding validation set.

split	ESM-2 weights	LoRA rank	LoRA alpha	LoRA dropout	learning rate	loss weight positive
0	facebook/esm2_t30_150M_UR50D	16	8	0.25	0.00001	10
1	facebook/esm2_t30_150M_UR50D	16	8	0.25	0.00001	10
2	facebook/esm2_t30_150M_UR50D	16	8	0	0.00001	3.162278
3	facebook/esm2_t30_150M_UR50D	16	8	0.25	0.00001	10
4	facebook/esm2_t12_35M_UR50D	64	8	0.25	0.000018	1

Supp. Table 3. Hyperparameters of the ablated TransFactor model without pre-trained weights with best AUROC in the corresponding validation set.

split	Transformer model dim	Transformer intermediate dim	# Transformer layer	# Attention heads	learning rate	loss weight positive
0	64	256	2	4	0.000056	3.162278
1	32	128	6	2	0.0001	10
2	128	512	4	8	0.000032	1
3	64	256	6	4	0.000056	1
4	32	128	2	2	0.000316	10

Supp. Table 4. Hyperparameters of the ablated TransFactor model with frozen ESM-2 with best AUROC in the corresponding validation set.

split	ESM-2 weights	learning rate	loss weight positive
0	facebook/esm2_t30_150M_UR50D	0.009914	10
1	facebook/esm2_t30_150M_UR50D	0.006033	3.162278
2	facebook/esm2_t30_150M_UR50D	0.001437	10
3	facebook/esm2_t30_150M_UR50D	0.000544	3.162278
4	facebook/esm2_t12_35M_UR50D	0.002384	10

Supp. Table 5. Hyperparameters of CNN-LSTM hybrid model with the best AUROC in the corresponding validation set.

split	# CNN filters	kernel size	max pool size	dropout	# CNN blocks	LSTM hidden size	learning rate	loss weight positive
0	1024	3	2	0	6	1024	0.000001	10
1	256	3	2	0	6	1024	0.000032	1
2	512	9	2	0.1	5	1024	0.000003	10
3	1024	3	2	0.05	6	512	0.00001	10
4	256	3	2	0.025	5	512	0.000018	10

Supp. Table 6. Hyperparameters of kmer SVM models, ideal thresholds determined on the corresponding validation set.

kernel	split	C	gamma	ideal threshold
RBF	0	0.3	scale	0.166627
RBF	1	0.3	scale	0.146367
RBF	2	0.3	scale	0.161599
RBF	3	0.3	scale	0.145732
RBF	4	0.3	scale	0.156187
linear	0	0.3	scale	0.136454
linear	1	0.3	scale	0.123095
linear	2	0.3	scale	0.134025
linear	3	0.3	scale	0.124325
linear	4	0.3	scale	0.128834

Supp. Table 7. Classification performance of experimental screens taken from the review of Baggen et al. (2021b). Similar to our benchmark, proteins were labeled as positives if they were functionally validated or detected in three or more studies, here without counting the study to be evaluated . The performance on the full proteome is shown. The table is sorted from lowest to highest F1-score.

**Reference**	**F1**	**Precision**	**Recall**
Baggen et al. (2021a)	0.02	0.41	0.01
Wang et al. (2021)	0.02	0.60	0.01
Wei et al. (2021)	0.04	0.78	0.02
Biering et al. (2021)	0.04	0.20	0.02
Hoffmann et al. (2021)	0.04	1.00	0.02
Zhu et al. (2021)	0.05	0.96	0.02
Daniloski et al. (2021)	0.06	0.53	0.03
Rebendenne et al. (2022)	0.06	0.33	0.03
Schneider et al. (2021)	0.06	0.44	0.04
Schmidt et al. (2021)	0.11	0.88	0.06
Davies et al. (2020)	0.11	0.79	0.06
Lee et al. (2021)	0.11	0.84	0.06
Kamel et al. (2021)	0.13	0.76	0.07
Labeau et al. (2022)	0.13	0.88	0.07
Li et al. (2021)	0.15	0.53	0.09
Flynn et al. (2021)	0.21	0.51	0.13
Gordon et al. (2020)	0.22	0.62	0.14
Stukalov et al. (2021)	0.24	0.34	0.19
Laurent et al. (2020)	0.25	0.20	0.34
Samavarchi-Tehrani et al. (2020)	0.37	0.29	0.52
St-Germain et al. (2020)	0.40	0.67	0.29
Mean±Standard deviation	0.13±0.11	0.60±0.25	0.11±0.13

Supp. Table 8. Performance of the same trained TransFactor models with predictions on proteins truncated to different maximum sequence lengths. For each truncated length, the first row reports the average performance and standard deviation across five individual models, each tuned and trained on a different fold. The second row presents the performance of an ensemble constructed from these five models. The same models are used across both truncated lengths; only the input differs.

Truncated length	AUROC	APS	F1-score	Precision	Recall	Precision@50	Precision@100	Precision@200
1024	0.87±0.01	0.27±0.01	0.25±0.12	0.40±0.34	0.39±0.27	0.37±0.03	0.32±0.02	0.26±0.01
	0.89	0.30	0.38	0.34	0.44	0.44	0.37	0.28
2048	0.88±0.01	0.28±0.03	0.28±0.14	0.31±0.12	0.44±0.27	0.38±0.03	0.32±0.02	0.27±0.03
	0.89	0.31	0.39	0.34	0.47	0.40	0.41	0.30

Supp. Table 9. Performance of TransFactor trained on SARS-CoV-2 host factors to predict SARS-CoV (N=612) (Stukalov et al., 2021) and HIV putative host factors (N=368) (Montoya et al., 2023) without further fine-tuning. The same threshold τ as used for SARS-CoV-2 was applied to obtain binary classes.

Pathogen	AUROC	APS	F1	Precision	Recall	P@50	P@100	P@200
SARS-CoV	0.80	0.11	0.19	0.12	0.42	0.16	0.19	0.20
HIV	0.64	0.03	0.05	0.03	0.18	0.06	0.05	0.03

C. Figures

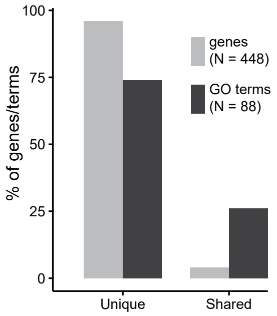

Supp Fig. 1: Related to Figure 2. The three functional studies from Baggen et al. (Flynn et al., Biering et al., Rebendenne et al.), with the highest amount of identified candidate host factors, were compared in terms of gene-wise overlap as well as in terms of overlap of enriched GO cellular compartment terms (reported by DAVID). Since these studies represent genome-wide screens, all human genes were used as background for GO enrichment. Terms with FDR-adjusted p-value < 0.05 were considered significant. Genes and GO-terms only significant in any one study are depicted as unique, while ones significant in two or more studies are considered shared.

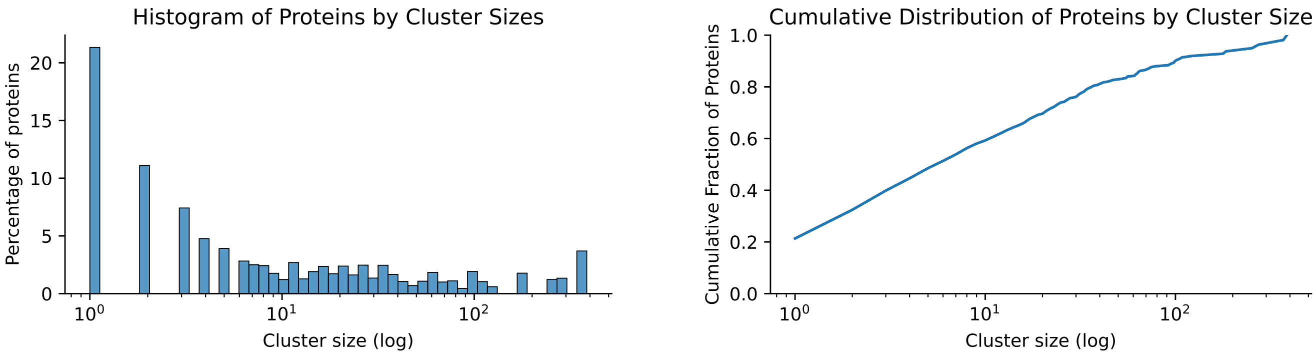

(a) (b)

Supp Fig. 2: Distribution of proteins by cluster size. Clusters were defined using mmseqs. a) Histogram of proteins by cluster sizes. b) Cumulative fraction of proteins belonging to cluster size.

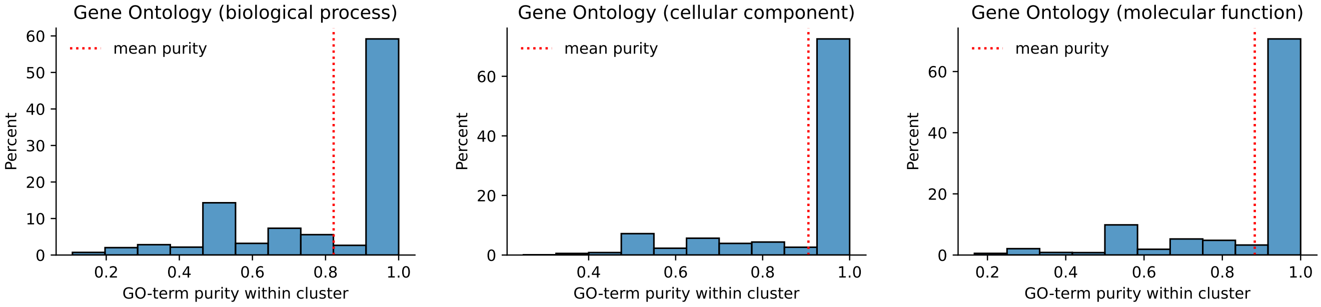

(a) (b) (c)

Supp Fig. 3: Purity of GO-terms within clusters defined by mmseqs. Proteins were annotated with their GO-term, and the fraction of proteins containing the most abundant term within a cluster is calculated for a) Biological Process, b) Cellular Component c) Molecular Function.

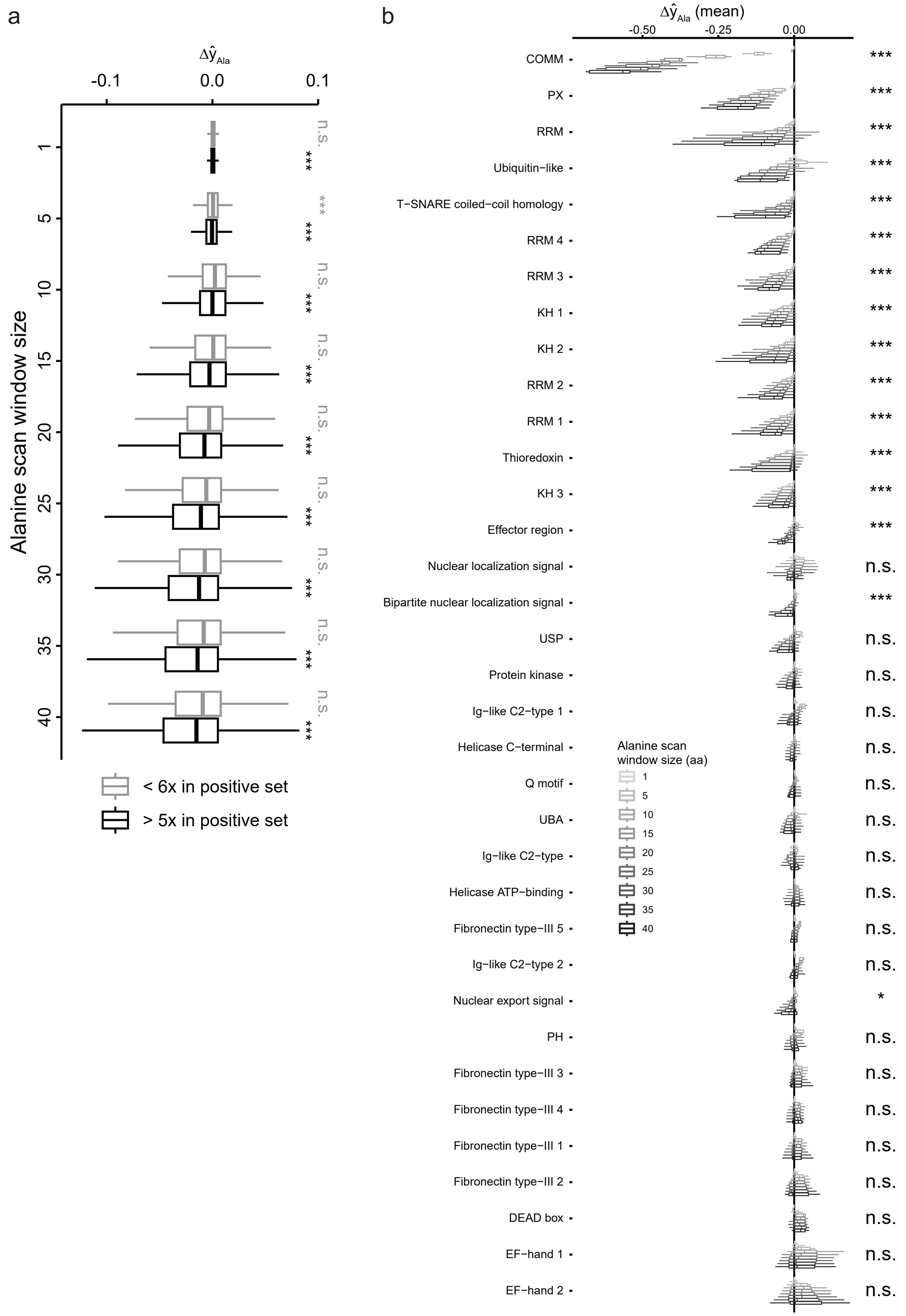

Supp Fig. 4: Related to Fig. 3b-g. Computational alanine scans were performed using indicated amino acid window sizes for all positively labeled proteins. (a) Domains, present more (black) or less or equal (grey) than 5 times in the positive samples, were used for the depiction of $\Delta\hat{y}_{Ala}$ distribution at indicated alanine scan window sizes. These distributions were further compared to the distribution of $\Delta\hat{y}_{Ala}$ across all positions at respective alanine scan window sizes. Statistical analysis was performed using the one-sided Wilcoxon rank-sum test, and the obtained p-values were further FDR-adjusted. (b) Distribution of mean $\Delta\hat{y}_{Ala}$ values within domains, present more than 5 times in the positive sample set, is depicted at the indicated alanine scan window sizes. $\Delta\hat{y}_{Ala}$ values for residues within these domains were compared to $\Delta\hat{y}_{Ala}$ across all scanned proteins using the one-sided Wilcoxon rank-sum test and alanine scan window size of 40. Thus obtained p-values were further FDR-adjusted. *** p.adj < 0.001, ** p.adj < 0.01, * p.adj < 0.05, n.s. (not significant) p.adj > 0.05.

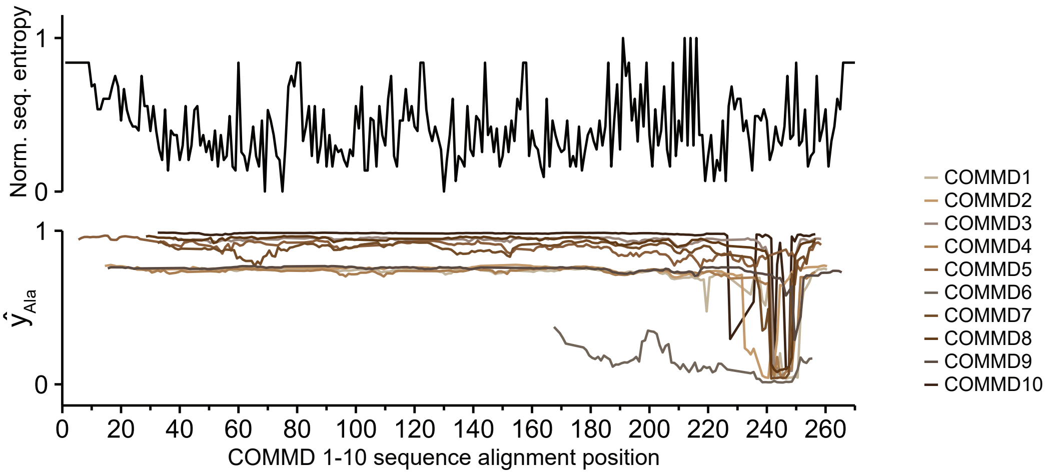

Supp Fig. 5: Related to Fig. 3j. Amino acid sequences of proteins COMMD1-10 were aligned using ClustalOmega ((Madeira et al., 2024)). Top: 10-letter amino acid alphabet normalized entropy (0 indicates low conservation, 1 indicates high conservation) of the alignment. Bottom: alanine scanning prediction values ($\hat{y}_{Ala}$) were corrected according to the depicted alignment and plotted for the indicated proteins (alanine scanning window size of 10).

References

J. Baggen, L. Persoons, E. Vanstreels, S. Jansen, D. Van Looveren, B. Boeckx, V. Geudens, J. De Man, D. Jochmans, J. Wauters, et al. Genome-wide crispr screening identifies tmem106b as a proviral host factor for sars-cov-2. *Nature genetics*, 53(4):435–444, 2021a.

J. Baggen, E. Vanstreels, S. Jansen, and D. Daelemans. Cellular host factors for sars-cov-2 infection. *Nature Microbiology*, 6(10): 1219–1232, 2021b.

S. B. Biering, S. A. Sarnik, E. Wang, J. R. Zengel, V. Sathyan, X. Nguyenla, E. Van Dis, C. Catamura, L. H. Yamashiro, A. Begeman, et al. Genome-wide, bidirectional crispr screens identify mucins as critical host factors modulating sars-cov-2 infection. *BioRxiv*, pages 2021–04, 2021.

A. Bressin, R. Schulte-Sasse, D. Figini, E. C. Urdaneta, B. M. Beckmann, and A. Marsico. Tripepsvm: de novo prediction of rna-binding proteins based on short amino acid motifs. *Nucleic acids research*, 47(9):4406–4417, 2019.

Z. Daniloski, T. X. Jordan, H.-H. Wessels, D. A. Hoagland, S. Kasela, M. Legut, S. Maniatis, E. P. Mimitou, L. Lu, E. Geller, et al. Identification of required host factors for sars-cov-2 infection in human cells. *Cell*, 184(1):92–105, 2021.

J. P. Davies, K. M. Almasy, E. F. McDonald, and L. Plate. Comparative multiplexed interactomics of sars-cov-2 and homologous coronavirus nonstructural proteins identifies unique and shared host-cell dependencies. *ACS infectious diseases*, 6(12):3174–3189, 2020.

R. A. Flynn, J. A. Belk, Y. Qi, Y. Yasumoto, J. Wei, M. M. Alfajaro, Q. Shi, M. R. Mumbach, A. Limaye, P. C. DeWeirdt, et al. Discovery and functional interrogation of sars-cov-2 rna-host protein interactions. *Cell*, 184(9):2394–2411, 2021.

D. E. Gordon, J. Hiatt, M. Bouhaddou, V. V. Rezelj, S. Ulferts, H. Braberg, A. S. Jureka, K. Obernier, J. Z. Guo, J. Batra, et al. Comparative host-coronavirus protein interaction networks reveal pan-viral disease mechanisms. *Science*, 370(6521):eabe9403, 2020.

S. Hochreiter. Long short-term memory. *Neural Computation MIT-Press*, 1997.

H.-H. Hoffmann, F. J. Sánchez-Rivera, W. M. Schneider, J. M. Luna, Y. M. Soto-Feliciano, A. W. Ashbrook, J. Le Pen, A. A. Leal, I. Ricardo-Lax, E. Michailidis, et al. Functional interrogation of a sars-cov-2 host protein interactome identifies unique and shared coronavirus host factors. *Cell host & microbe*, 29(2):267–280, 2021.

W. Kamel, M. Noerenberg, B. Cerikan, H. Chen, A. I. Järvelin, M. Kammoun, J. Y. Lee, N. Shuai, M. Garcia-Moreno, A. Andrejeva, et al. Global analysis of protein-rna interactions in sars-cov-2-infected cells reveals key regulators of infection. *Molecular cell*, 81(13): 2851–2867, 2021.

A. Labeau, L. Fery-Simonian, A. Lefevre-Utile, M. Pourcelot, L. Bonnet-Madin, V. Soumelis, V. Lotteau, P.-O. Vidalain, A. Amara, and L. Meertens. Characterization and functional interrogation of the sars-cov-2 rna interactome. *Cell reports*, 39(4), 2022.

E. M. Laurent, Y. Sofianatos, A. Komarova, J.-P. Gimeno, P. S. Tehrani, D.-K. Kim, H. Abdouni, M. Duhamel, P. Cassonnet, J. J. Knapp, et al. Global bioid-based sars-cov-2 proteins proximal interactome unveils novel ties between viral polypeptides and host factors involved in multiple covid19-associated mechanisms. *BioRxiv*, pages 2020–08, 2020.

S. Lee, Y.-s. Lee, Y. Choi, A. Son, Y. Park, K.-M. Lee, J. Kim, J.-S. Kim, and V. N. Kim. The sars-cov-2 rna interactome. *Molecular cell*, 81(13):2838–2850, 2021.

J. Li, M. Guo, X. Tian, X. Wang, X. Yang, P. Wu, C. Liu, Z. Xiao, Y. Qu, Y. Yin, et al. Virus-host interactome and proteomic survey reveal potential virulence factors influencing sars-cov-2 pathogenesis. *Med*, 2(1):99–112, 2021.

F. Madeira, N. Madhusoodanan, J. Lee, A. Eusebi, A. Niewielska, A. R. Tivey, R. Lopez, and S. Butcher. The embl-ebi job dispatcher sequence analysis tools framework in 2024. *Nucleic Acids Research*, page gkae241, 2024.

V. R. Montoya, T. M. Ready, A. Felton, S. R. Fine, M. OhAinle, and M. Emerman. A virus-packageable crispr system identifies host dependency factors co-opted by multiple hiv-1 strains. *Mbio*, 14(1):e00009–23, 2023.

A. Rebendenne, P. Roy, B. Bonaventure, A. L. Chaves Valadão, L. Desmarets, M. Arnaud-Arnould, Y. Rouillé, M. Tauziet, D. Giovannini, J. Touhami, et al. Bidirectional genome-wide crispr screens reveal host factors regulating sars-cov-2, mers-cov and seasonal hcovs. *Nature genetics*, 54(8):1090–1102, 2022.

P. Samavarchi-Tehrani, H. Abdouni, J. D. Knight, A. Astori, R. Samson, Z.-Y. Lin, D.-K. Kim, J. J. Knapp, J. St-Germain, C. D. Go, et al. A sars-cov-2–host proximity interactome. *BioRxiv*, pages 2020–09, 2020.

N. Schmidt, C. A. Lareau, H. Keshishian, S. Ganskih, C. Schneider, T. Hennig, R. Melanson, S. Werner, Y. Wei, M. Zimmer, et al. The sars-cov-2 rna–protein interactome in infected human cells. *Nature microbiology*, 6(3):339–353, 2021.

W. M. Schneider, J. M. Luna, H.-H. Hoffmann, F. J. Sánchez-Rivera, A. A. Leal, A. W. Ashbrook, J. Le Pen, I. Ricardo-Lax, E. Michailidis, A. Peace, et al. Genome-scale identification of sars-cov-2 and pan-coronavirus host factor networks. *Cell*, 184(1): 120–132, 2021.

J. R. St-Germain, A. Astori, P. Samavarchi-Tehrani, H. Abdouni, V. Macwan, D.-K. Kim, J. J. Knapp, F. P. Roth, A.-C. Gingras, and B. Raught. A sars-cov-2 bioid-based virus-host membrane protein interactome and virus peptide compendium: new proteomics resources for covid-19 research. *BioRxiv*, pages 2020–08, 2020.

A. Stukalov, V. Girault, V. Grass, O. Karayel, V. Bergant, C. Urban, D. A. Haas, Y. Huang, L. Oubraham, A. Wang, et al. Multilevel proteomics reveals host perturbations by sars-cov-2 and sars-cov. *Nature*, 594(7862):246–252, 2021.

R. Wang, C. R. Simoneau, J. Kulsuptrakul, M. Bouhaddou, K. A. Travisano, J. M. Hayashi, J. Carlson-Stevermer, J. R. Zengel, C. M. Richards, P. Fozouni, et al. Genetic screens identify host factors for sars-cov-2 and common cold coronaviruses. *Cell*, 184(1):106–119, 2021.

J. Wei, M. M. Alfajaro, P. C. DeWeirdt, R. E. Hanna, W. J. Lu-Culligan, W. L. Cai, M. S. Strine, S.-M. Zhang, V. R. Graziano, C. O. Schmitz, et al. Genome-wide crispr screens reveal host factors critical for sars-cov-2 infection. *Cell*, 184(1):76–91, 2021.

S. Wu and J.-t. Guo. Improved prediction of dna and rna binding proteins with deep learning models. *Briefings in Bioinformatics*, 25 (4), 2024.

Y. Zhu, F. Feng, G. Hu, Y. Wang, Y. Yu, Y. Zhu, W. Xu, X. Cai, Z. Sun, W. Han, et al. A genome-wide crispr screen identifies host factors that regulate sars-cov-2 entry. *Nature communications*, 12(1):961, 2021.
